# Optimization of Taste-Masked (–)-Oleocanthal Effervescent Formulation with Potent Breast Cancer Progression and Recurrence Suppressive Activities

**DOI:** 10.3390/pharmaceutics11100515

**Published:** 2019-10-05

**Authors:** Afsana Tajmim, Abu Bakar Siddique, Khalid El Sayed

**Affiliations:** School of Basic Pharmaceutical and Toxicological Sciences, College of Pharmacy, University of Louisiana at Monroe, 1800 Bienville Drive, Monroe, Louisiana 71209, USA; tajmima@warhawks.ulm.edu (A.T.); siddiqab@warhawks.ulm.edu (A.B.S.)

**Keywords:** oleocanthal, effervescent formulation, breast cancer, taste masking, recurrence, HER2

## Abstract

*S*-(–)-Oleocanthal (OC), a naturally occurring phenolic secoiridoid exclusively found in extra-virgin olive oil (EVOO), is a potential nutraceutical therapeutic for inflammation, neurodegenerative diseases, and many malignancies, especially breast cancer (BC). The oral delivery of OC is challenging because of its irritative, bitter, and pungent taste and exceptional chemistry, including two reactive aldehydes, phenolic, and ester groups. OC irritation did not correlate with CO_2_-induced irritation, and hence, OC was not exerting generalized acid-sensing irritation. The objective of this study was to develop an effervescent formulation of OC with an effective CO_2_-induced masked taste maintaining the efficacy against the estrogen receptor (ER) and HER2 positive BC. Several ratios of acid and carbonate sources were screened, and five effervescent formulations EF1-EF5 were selected and prepared based on their pH and effervescence time. OC formulations were characterized using differential scanning calorimetry, FT-IR spectroscopy, and scanning electron microscopy analyses. OC formulations exhibited acceptable flowability and effervescence time. Based on physical characteristics and improved OC release, formulation EF-2 was selected for subsequent studies. EF-2 showed effective OC taste masking, as suggested by electronic artificial tongue and mouse preference tests. EF-2 suppressed more than 70% of the hormone and HER2-positive BT-474 BC cell growth in a nude mouse xenograft model. Furthermore, EF-2 demonstrated significant inhibition of BT-474 tumor cell locoregional recurrence after primary tumor surgical excision. EF-2-treated mouse sera had significantly reduced CA 15-3 levels, the human BC recurrence marker, compared to the placebo control group at the end of the study. These results highlight the potential of the OC formulation EF-2 as a prospective nutraceutical for the control and prevention of ER^+^/HER^+^ BC progression and locoregional recurrence.

## 1. Introduction

Extra-virgin olive oil (EVOO) is one of the key components in the Mediterranean diet. It has been pointed as a contributing factor towards the epidemiologically documented favorable health benefits in Mediterranean populations due to the presence of minor phenolic ingredients of EVOO [1,2]. EVOO has gained notable scientific attention focused on the beneficial effects of its phenolic components, including anti-inflammatory, antioxidant, and antimicrobial activities. Several studies have already shown the correlation of a high intake of EVOO with a lower incidence of colon and breast cancers, osteoporosis, cardiovascular, metabolic, and Alzheimer’s disease [3,4,5].

(–)-Oleocanthal (OC, decarboxymethyl ligstroside aglycone) is a unique phenolic secoiridoid exclusively occurring in EVOO. OC provides the irritative, bitter, pharyngeal pungent, and astringent taste of EVOO [6,7]. OC was first discovered in 1992, and its chemical structure was reported later [8,9]. In addition, in 2005, the Beauchamp group suggested the name oleocanthal with the suffix “*oleo*” for olive, “*canth*” for stinging, and “*al*” for its aldehydes, and reported its potent anti-inflammatory activity [10]. Several literatures later documented the anti-inflammatory, antioxidant, antimicrobial, anticancer, and neuroprotective activities of OC [1].

The anti-inflammatory potency of OC was comparable with the non-steroidal anti-inflammatory drugs (NSAID) like ibuprofen by inhibiting COX-1 and COX-2. OC also exhibited an irritant taste similar to ibuprofen [10,11]. The OC dialdehydic functionality was found to be the main pharmacophore activating the transient receptor potential cation channel subtype A1 (TRPA1) receptor, which translates the irritative and pungent taste sensation [12,13]. Distinct OC nociceptors were suggested in the oral cavity-oropharyngeal region.

OC inhibited the LPS-mediated upregulation of proinflammatory signaling molecules, including interleukin-1β (IL-1β), IL-6, macrophage inflammatory protein-1α (MIP-1α), tumor necrosis factor-α (TNF-α), and granulocyte-macrophage-colony-stimulating factor (GM-CSF). OC showed neuroprotective effects against Alzheimer’s disease by altering the structure and function of β-amyloids, tau phosphorylation, and reducing the inflammation of astrocytes [14,15,16,17]. On the other hand, numerous studies showed that OC played a role in inducing apoptosis and inhibiting the migration, angiogenesis, and metastasis of cancerous cell lines originating from hepatocellular carcinoma [18], prostate cancer [19], human melanoma [20], and non-melanoma skin cancers [21], colorectal carcinoma [22], and breast cancer (BC) [7,23]. OC exhibited its anti-BC and anti-prostate cancer effects through competitive inhibition of c-MET kinase ATP activation [7,19]. OC acted via AMPK inhibition by the suppression of MIP-1α in multiple myeloma [22].

Despite the documented in vitro and in vivo bioactivities, there is no reported pharmaceutically acceptable oral dosage form of OC to date. Thus, an acceptable OC pharmaceutical oral dosage form is needed to facilitate its application as a potential nutraceutical for diverse therapeutic applications. On the other hand, appropriate OC oral formulation must carefully consider its chemical instability, due to the reactive aldehyde and ester groups, irritative and pungent taste, and poor water solubility, and maintain its in vivo bioactive potency. The effervescent composition is a convenient dosage form producing CO_2_ effervescence as an underused formulation approach in which the Active Pharmaceutical Ingredient (API) is administered in aqueous solution form. CO_2_ effervescence imparts taste masking of the pungent and bitter taste of APIs [24]. Interestingly, the OC irritative taste was not correlated with the acid-induced irritation, and hence, OC was proven not to exert generalized acid-sensing irritation [13]. The taste intensity ratings of OC and CO_2_ were not correlated, and therefore, they were hypothesized to have independent irritative taste mechanisms [13]. This may facilitate the use of CO_2_ for effective OC taste masking. Furthermore, the effervescence composition is obtained by reacting acids like citric and/or tartaric acids with bases like carbonates or bicarbonates in the presence of water, which releases CO_2_ to provide acceptable carbonated or sparkling drinkable liquid. Due to the liberation of CO_2_, the dissolution is expected to improve by the imparted acidity [25]. In addition, the effervescent formulation provides functional and consumer advantage over conventional pharmaceutical dosage forms. Therefore, effervescent formulations are favored by geriatric and pediatric patient populations, in addition to adult populations with gag reflexes and/or swallowing difficulty [26].

Thus, the main objectives of this study are: (i) to develop an effervescent OC dosage form with effective taste masking and enhanced dissolution; (ii) to assess the efficacy of the formulated effervescent powder against the growth of the estrogen receptor positive/human epidermal receptor-2 positive (ER^+^/HER2^+^) BC in vitro and in vivo in nude mouse xenograft models; (iii) to determine the ER^+^/HER2^+^ BC locoregional recurrence inhibitory efficacy of the formulated effervescent powder in an orthotopic xenograft mouse model after primary tumor surgical excision.

## 2. Materials and Methods

### 2.1. Chemicals, Reagents, and Antibodies

Citric acid, tartaric acid, and sodium bicarbonate were purchased from Fisher Scientific (New Brunswick, NJ, USA). In addition, mannitol and aerosil-200 were acquired from Sigma Aldrich (St. Louis, MO, USA). All primary and secondary antibodies were purchased from Cell Signaling Technology (Beverly, MA, USA), unless otherwise stated. The CA 15-3 (Human) ELISA Kit (Catalog number KA0206) was purchased from Abnova (Walnut, CA, USA).

### 2.2. (–)-Oleocanthal Isolation from Extra-Virgin Olive Oil Samples

Oleocanthal was extracted from EVOO (The Governor, batch #: 5-214000-242017) using a liquid–liquid extraction method where the successful capacity of water was used to efficiently extract OC as its monohydrate and utilize its self-emulsifying tendency [26]. Selective resin entrapment enabled the water and residual fatty acids elimination and recovered OC in high-yield and good purity [26]. Final purification was conducted on Sephadex LH20, isocratic elution with CH_2_Cl_2_. The purity of OC (>99%) was assessed on a Phenomenex Cosmosil 5C18-AR-II column (250 mm × 4.6 mm, 5 µm; Phenomenex Inc., Torrance, CA, USA) using 1:1 H_2_O–CH_3_CN isocratic elution as a mobile phase. The purity and chemical identity of OC was further confirmed by NMR analysis on a JEOL Eclipse ECS-400 NMR spectrometer. The q^1^H NMR confirmed >99% purity of OC, which was stored at −20 °C in a freezer in an amber glass vial after purging out air with N_2_ gas [26].

### 2.3. Determination of Effervescent Components

The effervescent ingredients and their ratios were optimized depending on acid–base interaction. Different amounts of citric acid, tartaric acid, and sodium bicarbonate were mixed and poured into 100 mL of water, followed by the immediate measurement of effervescence time and pH. The effervescence time and pH were evaluated by using a standard stopwatch and calibrated pH meter (pH meter, OAKTON Instruments, Vernon Hills, IL, USA), respectively. Each experiment was repeated three times.

### 2.4. Preparation of Effervescent Powder

OC was mixed with flavor and then adsorbed by the adsorbing agent Aerosil 200 (Mixture-1). On the other hand, the required quantities of citric acid, sodium bicarbonate, and tartaric acid were uniformly blended together. Mannitol was then added, mixed with the blended powder of acids and sodium bicarbonate, and passed through a 40-mesh screen (mixture-2). Mixture-2 was added to mixture-1 in different ratios, thoroughly blended, and passed through a 40-mesh screen. Formulation randomization was based on various ratios of citric, tartaric, and NaHCO_3_ contents. One ingredient was fixed, and others optimized in various ratios to create various formulations. Different formulations were prioritized based on the optimal effervescent time and pH results.

### 2.5. Determination of Solution pH

Each effervescent formulation powder was dissolved into 300 mL of deionized distilled water, and the pH of the solution was measured by using a pH meter. Each experiment was repeated three times.

### 2.6. Determination of Effervescence Time

Each effervescent formulation powder was poured into 300 mL of deionized distilled water, and in vitro effervescence time was determined by a stopwatch. Each experiment was repeated three times.

### 2.7. Determination of CO_2_ Content

A glass beaker containing 80 mL of deionized distilled water was covered with a polyethylene cover, which was cut in a rectangular shape (2.9 × 0.6 cm) so that the effervescent powder easily passed through the beaker [27]. This covered glass beaker was kept on an analytical balance at room temperature and the weight was recorded. A total of 1 g of each effervescent formulation powder was dropped into the beaker and left for 2 min. The final weight loss was recorded, representing the CO_2_ amount in mg lost during the effervescent reaction.

### 2.8. Flow Properties of Powder

#### 2.8.1. Angle of Repose (*θ*)

The static angle of repose was determined by the fixed funnel method, which is exhibited as the maximum possible angle between the surface of a powder pile or powder and the horizontal plane. The powder was allowed to pass through a funnel fixed to a stand at a definite height. Each experiment was repeated three times. The angle of repose (*θ*) was calculated by measuring the height (*h*) and radius (*r*) of the formed powder heap using the formula [28]:

tan (*θ*) = (*h*/*r*).

#### 2.8.2. Compressibility Index

The flowability of powder was measured by comparing the powder bulk density (*D*p) and tapped density (*D*t). Each experiment was repeated three times. The compressibility index percentage was calculated using the formula:

Carr’s index = ((*D*p − *D*t)/*D*p) × 100.

#### 2.8.3. Hausner’s Ratio

Hausner’s ratio is an important property to determine the flow property of powder. Each experiment was repeated three times. This can be calculated using the following formula:

Hausner’s ratio = *D*p/*D*t,

with bulk density (*D*p) and tapped density (*D*t) of powder.

### 2.9. Fourier Transform Infrared Spectroscopy

The Fourier transform infrared (FT-IR) spectra of all formulations, plain non-formulated OC, and placebo carrier were recorded using a PerkinElmer Spectrum-Two™ FT-IR spectrometer (Waltham, MA, USA). Samples were analyzed using a diffuse reflectance cell, without prior sample preparation, by directly compressing on the ATR crystal under appropriate compression conditions and scanned at a resolution of 4 cm^−1^ using the absorbance over the wavenumber range from 400–4000 cm^−1^. Each sample IR spectrum was acquired three times.

### 2.10. Thermal Analysis of the Effervescent Powder by Differential Scanning Calorimetry (DSC)

The thermal analysis of each of the five effervescent powders was performed using a TA 2920 modulated differential scanning calorimeter (DSC, TA Instruments-Waters LLC, New Castle, DE, USA) under nitrogen atmosphere at a flow rate of 25 mL/min. An accurately weighed 5 mg of each sample was hermetically sealed in an aluminum crimp pan and heated from 0–350 °C at a temperature acceleration rate 10 °C/min. The sample was cooled using a DSC refrigerated cooling system (TA Instruments). Melting endotherms were analyzed by the universal analysis 2000 software, V 4.2 (TA Instruments-Waters LLC, New Castle, DE, USA) and compared with the placebo.

### 2.11. Scanning Electron Microscopy (SEM) and Confocal Laser Scanning Microscopy (CLSM)

EF-2 was placed on a brass stub with the application of double-sided adhesive tape, and then coated in a vacuum chamber with a thin layer of gold for 30 seconds (Sputter coater, Edwards, S150A, England, UK) to prepare it as electrically conductive. The pictures were taken at an excitation voltage of 20 kV (Electron probe microanalyzer, JEOL, JXA-840A, Tokyo, Japan) to check the surface morphology of the selected formula. A confocal laser scanning microscope was used to examine the surface morphology of EF-2. A Biorad MRC 1024 Laser Scanning Confocal Imaging System (Hemel Hempstead, UK), equipped with an argon ion laser (American Laser Corp, Salt Lake City, UT, USA) and a Zeiss Axiovert 100 microscope (Carl Zeiss, Oberkochen, Germany) was used to examine the effervescent formulation.

### 2.12. In-Vitro Dissolution Study

The dissolution profile of each of plain non-formulated OC and EF-2 powder formulation was carried out in a 100 mL simulated gastric fluid (SGF, pH 1.2), without enzymes, and simulated intestinal fluid (SIF, pH 6.8), without enzymes. This test was conducted by utilizing a USP type II dissolution apparatus (VK 7000, Varian Inc., Cary, NC, USA) at a paddle speed of 100 rpm. The temperature of the dissolution medium was maintained at 37 ± 5 °C using a Varian VK750 heater (Varian Inc., Cary, NC, USA). The effervescent powder of 10 mg of OC was packed into a size 00 transparent hydroxypropyl methylcellulose (HPMC) capsule and inserted into the medium by using an individual sinker. About 1 mL aliquot sample was withdrawn at time intervals of 2, 5, 10, 20, 30, 40, and 60 min, which was replaced with an equivalent amount of fresh dissolution medium. The collected samples were filtered, and OC content was analyzed by an HPLC system equipped with a UV/Visible variable wavelength detector at *λ*_max_ 230 nm (Shimadzu Scientific Instrument, Japan). Each experiment was repeated three times. A 20 μL sample was then injected into the Eclipse YD5 C18-RP analytical column (4.6 mm × 15 cm) at a flow rate of 1.0 mL/min. Acetonitrile and water (50:50) mixture was used isocratically as a mobile phase. Data acquisition and analysis were performed using Lab Solution™ chromatography software.

### 2.13. Taste Assessment Using an Electronic Tongue

The ASREE electronic tongue, generated by Alpha MOS, is an adequate tool for assessing taste sensing and therefore used to compare the effectiveness of effervescent formulation to mask the irritant OC taste with OC and a placebo. This test measures the comparative percentage between two principal component analyses (PCA1-PCA2) to describe total data variation by processing data acquired by the e-tongue from seven different taste sensors to two-dimensional data. The assays were analyzed on the ASTREE e-tongue system equipped with the array consisting of an Alpha MOS sensor set #2 for pharmaceutical analysis, which composed of 7 specific sensors (ZZ, AB, GA, BB, CA, DA, JE) [29,30]. The system composed of a 48-position autosampler and 25 mL capacity beakers for sampling, an array of liquid sensors, an electronic unit for sensor data acquisition using multidimensional chemometric statistics-Alpha Soft v15.0 software, and the acquisition times were fixed at 120 seconds. The euclidian distances and percentage discrimination index (DI) between samples were calculated to assess taste proximity; the lower the distance, the closer the taste. Data generated by the ASTREE system processed taste analysis was conducted in artificial saliva without enzymes, pH 6.8; X1, comparing a vehicle control, non-formulated OC; X2, effervescent formulation; X3, placebo formulation; X4. Discrimination index (DI in %) was determined for each formulation and placebo pair. The closer the DI to 100%, the greater the distance between the centers of gravity and the smaller the dispersion within groups.

### 2.14. Animal Preference Experiment

Male and female 6-week old Swiss Albino mice weighing 20–25 g were obtained from Envigo (Indianapolis, IN, USA). All animal experiments were approved by the Institutional Animal Care and Use Committee (IACUC), University of Louisiana at Monroe, protocol number 17OCT-KES-01, approved on 17 October 2017, with good animal practice defined by the NIH guidelines. Mice were acclimated to the University of Louisiana-Monroe, College of Pharmacy animal housing facility and maintained under clean room conditions in sterile filter top cages using Alpha-Dri bedding and high efficiency particulate air-filtered ventilated plastic racks at 25 °C, 55–65% relative humidity, and a 12 h light/dark cycle for a week before experiments. Mice had free access to purified drinking water and pelleted rodent chow (no. 7012, Envigo/Teklad, Madison, WI, USA). Taste preferences were assessed for 48 h using a two-bottle choice test [31] for the following samples:

(i) Plain, non-formulated OC, (ii) effervescent formulation EF-2 at a daily oral dose of 10 mg OC/kg of body weight, and (iii) placebo formulation. A total of 30 mice (15 males and 15 females) were randomized into 3 groups for each gender, *n* = 5 mice. The mice had access to two drinking bottles; one contained distilled water, and the other contained either a plain non-formulated OC sample, EF-2, or placebo formulation for 48 h. The positions of both drinking bottles were switched every 24 h. The volume consumed of each bottle was recorded (using a volumetric level scale to the nearest 0.1 mL) at the beginning and end of the experiment after 48 h. The total fluid intake of each sample was obtained by adding the volume intakes for each sample. Percentage preference was calculated as intake of the solution of sample bottle divided by the total fluid intake.

### 2.15. Cell Lines and Culture Conditions

The human BC cell lines MDA-MB-231 and BT-474 were purchased from ATCC and maintained in RPMI-1640 supplemented with 10% FBS, 100 U/mL penicillin, 0.1 mg/mL streptomycin in a humidified atmosphere of 5% CO_2_ at 37 °C. All cells were maintained at 37 °C in an environment of 95% air and 5% CO_2_ in a humidified incubator.

MDA-MB-231 or BT-474 cells were plated at a density of 1 × 10^4^ cells per well (6 wells/group) in 96-well culture plates and maintained in RPMI-1640 media supplemented with 10% FBS, which allowed to adhere overnight. The next day, cells were washed with phosphate buffer saline (PBS), divided into different treatment groups and then given various concentrations of EF-2 formulation or placebo treatment media for 48 h. Viable cells count was determined using the 3-(4,5-dimethylthiazol-2yl)-2,5-diphenyl tetrazolium bromide (MTT) colorimetric assay, whereas the optical density of each sample was measured at 570 nm on a microplate reader (BioTek, VT, USA). Furthermore, the number of cells/well was calculated against a standard curve prepared by plating various concentrations of cells, which were measured by using a hemocytometer at the beginning of each experiment [32].

### 2.16. Effects of EF-2 on BT-474 Nude Mice Tumor Xenograft Progression and Recurrence Models

The inhibitory effects of EF-2 formulation administration against the growth and recurrence of the human HER2^+^-ER^+^ BC cells - BT-474 cells orthotopically xenografted in nude mice were assessed. Foxn1^nu^/Foxn1^+^, 4–5 week old, female athymic nude mice were purchased from Envigo (Indianapolis, IN). All animal experiments were approved by the Institutional Animal Care and Use Committee (IACUC), University of Louisiana at Monroe, protocol number 18 MAY-KES-02, approved on 18 May 2018, with good animal practice defined by the NIH guidelines. Mice were acclimated to the University of Louisiana-Monroe, College of Pharmacy animal housing facility and maintained under clean room conditions in sterile filter top cages using Alpha-Dri bedding and high efficiency particulate air-filtered ventilated racks at 25 °C, 55–65% relative humidity, and a 12 h light/dark cycle for a week before experiments. Husk and excreta were taken away from the cages daily. Mice had free access to purified drinking water and pelleted rodent chow (no. 7012, Envigo/Teklad, Madison, WI). Animals were orally dosed daily at 10 mg/kg OC in EF-2 dissolved in sterile normal saline using 18G plastic (PTFE) with stainless steel bite protector oral feeding needles (VWR, Suwanee, GA, USA).

#### 2.16.1. Tumor Growth Inhibition

BT-474 human BC cells were cultured and resuspended in serum-free RPMI-1640 medium and Matrigel with a 50:50 ratio. In addition, after anesthesia, cell suspensions (5 × 10^6^ cells/60 µL) were subcutaneously inoculated into the second mammary gland fat pad just beneath the nipple of each animal to generate orthotopic breast tumors. Mice were then randomly divided into two groups: i) the placebo control group (*n* = 5), and ii) the EF-2-treated group (*n* = 5), at a dose of 10 mg OC/kg. Oral treatments, placebo control, or EF-2 started on the tumor cells inoculation day and continued daily thereafter. EF-2 was dissolved in 1 mL of water at a concentration of 1 mg/mL and immediately given fresh to the mice every day. The mice were monitored daily by measuring tumor volume, body weight, and clinical observation. Tumor volume (V) was calculated by V = L/2 × W^2^, where L was the length and W was the width of tumors. At the end of the experiment, the primary tumors were surgically excised and weighed.

#### 2.16.2. Tumor Recurrence Inhibition

To investigate the efficacy of EF-2 against recurrence tumor, animals used in the previous growth models were used. Once the average tumor volume in the control mice group reached ~1000 mm^3^, mostly on day 27, animals were anesthetized with *i.p.* ketamine/xylazine combination (100 mg/kg / 15 mg/kg) and their primary tumors were surgically excised. Each animal surgery wound was aseptically closed by one or two stitches. Ketoprofen, 1 mg/kg, was used 12 h before and after surgery for effective analgesia. Ophthalmic lubricant was used during the surgery to prevent corneal drying. Bupivicaine (0.25%, 1–2 drops), twice daily, was used topically at the excision wound site, local infiltration along the surgery site during closure with a maximum dose of 2 mg/kg. One day after surgery, mice maintained previous treatment groups: (i) the placebo control group (*n* = 5), (ii) the EF-2-treated group (*n* = 5). Treatments continued for an additional 30 days. The mice were routinely monitored by measuring tumor volume, body weight, and clinical observations. These observations included daily monitoring of general mice health characters (food/water intake, body weight, hydration status, defecation, urination, physical activity, and behavior). Wound incisions were carefully examined daily to ensure that wounds were contamination and inflammation-free, clean, and dry. All mice were then sacrificed, and individual tumors were excised, collected, and weighed. The results presented as average ± SD.

### 2.17. Western Blot Analysis

Collected breast tumor tissues after animal sacrifice were stored at −80 °C until protein extraction. To make sure every animal in each experimental group was represented in the western blotting results, equal parts of each collected breast tumor tissues were combined for each treatment group and homogenized in RIPA buffer (Qiagen Sciences Inc., Valencia, CA, USA) using an electric homogenizer. The protein concentration was then determined by the BCA assay (Bio-Rad Laboratories, Hercules, CA, USA). Equivalent amounts of protein were electrophoresed on SDS–polyacrylamide gels, whereas the gels were electroblotted onto PVDF membranes. Furthermore, these PVDF membranes were blocked with 2% BSA in 10 mM Tris-HCl containing 50 mM NaCl and 0.1% Tween-20, pH 7.4 (TBST), and then incubated with specific primary antibodies overnight at 4 °C according to the manufacturer protocol. At the end of the incubation period, membranes were washed five times with TBST and then incubated with respective horseradish peroxide-conjugated secondary antibody in 2% BSA in TBST for 1 h at room temperature, followed by rinsing with TBST five times. Blots were then visualized by chemiluminescence according to the manufacturer’s instructions (Pierce, Rockford, IL, USA). Proteins were detected using the ChemiDoc XRS chemiluminescent gel imaging system and analyzed using Image Lab software (Bio-Rad Laboratories). Here, visualization of β-tubulin was used to ensure equal sample loading in each lane. All experiments were repeated three times [33].

### 2.18. Evaluation of EF-2 Treatment on the Human Serum CA 15-3 Biomarker Level

The cancer antigen 15-3 (CA 15-3) is a specific tumor marker usually used to monitor the response of the BC treatment and predict the potential of recurrence. Mice serum samples were collected at the end of the recurrence experiment and used for the determination of the concentration of the CA 15-3 without any modification. About 20 µL of serum sample was mixed with 1.0 mL of sample diluent, following the manufacturer protocol (Abnova, Catalog Number KA0206). About 200 µL of CA 15-3 standards, diluted specimens, and diluted controls were added into the appropriate wells and gently mixed for 10 s and incubated at 37 °C for 1 h, and the microtiter plate was rinsed and emptied 5 times by washing buffer (1×). Nearly 200 µL of enzyme conjugate reagent was dispensed into each well and gently mixed for 10 s followed by incubation at 37 °C for 1 h. In addition, 100 µL of TMB reagent was dispensed into each well and gently mixed for 10 s, followed by incubation at room temperature in the dark for 20 min. Finally, the reaction was stopped by adding 100 µL of stopping solution to each well and gently mixing for 30 s. Absorbance was read immediately at an optical density of 450 nm with a microtiter plate reader at the end of the experiment [33].

### 2.19. Statistical Analysis

Values were expressed as mean ± standard deviation (SD) and differences among placebo and EF-2-treated groups followed by the analysis of unpaired *t*-test using GraphPad Prism version 8. Animal preference test was analyzed by One-way ANOVA followed by Tukey’s test. A difference of *p* < 0.05 was considered statistically significant as compared to the placebo control group.

## 3. Results

### 3.1. Determination of Ph, Effervescence Time, and CO_2_ Content of OC Effervescent Formulations

The CO_2_ gas generation reaction of the effervescent powder is due to the water dissolution and initiating reaction of acids (citric and tartaric acids) with sodium bicarbonate. The liberation rate of CO_2_ is proportional to the water volume. Hence, selecting anhydrous, water-free, or stable hydrate raw materials and excipients are preferred [27]. The combination of citric acid and tartaric acid was optimized based on the effervescence time and pH of the combination. An array of citric acid-tartaric acid combination ratios was attempted (P1–P27, Table 1).

The use of a single acid did not provide enough effervescence time and desired pH, which justified the combination optimization. Combined excipients provided better effervescence time and pH versus a single acid excipient. The use of individual citric acid resulted in a sticky formulation mixture, whereas using tartaric acid alone rendered the formulation to a crumbled powder without mechanical strength. Combining both acids overcame these issues and offered a physically acceptable mixture. Once initial effervescent excipients were established, optimization resulted in five different effervescent formulations EF-1-EF-5 (Table 2). Table 3 lists the pH, effervescence time, and CO_2_ content of different effervescent powder formulations. Each effervescent powder was dissolved in water to provide a clear solution, and then the pH was measured at a specific time. The pH of the effervescent solution can change on standing. Consistent assessment of effervescent solution pH is a predictor of uniform distribution of raw materials within the formulation.

EF-1-EF-5 were selected based on the pH range and effervescent time (Table 3). Among five different formulations, EF-1, EF-3, and EF-4 showed a pH range of 3.21–3.95 (Table 3), which can be too acidic and can cause gastro-intestinal (GI) irritation. On the other hand, EF-2 showed a pH of 4.86 (Table 3), an optimal acidic range which can facilitate OC irritative taste masking and provide better palatability without causing GI irritation. Ideal effervescence time optimized to be <3 min [34]. The effervescence time of all formulations met this specification (Table 3). CO_2_ formation drives the effervescence, and therefore, the quality and efficiency of effervescence formulations had been constantly monitored by measuring CO_2_ content (Table 3). EF-2 provided the highest CO_2_ content in comparison with other formulations (Table 3).

### 3.2. Flow Properties of Effervescent Powder

Flow properties are important to assess the quality of powdered pharmaceuticals. The angle of repose, compressibility index, and Hausner’s ratio of all formulations are shown in Table 4. EF-1 and EF-4 showed the angle of repose of 33.82° ± 2.87° and 33.82° ± 3.01°, respectively, which suggested good flow properties. Meanwhile, EF-2, EF-3, and EF-5 showed the angle of repose of 28.68° ± 3.14°, 25.64° ± 1.87°, and 20.80° ± 2.43°, respectively, which possessed excellent flow properties. The compressibility index of EF-2 was 5.00% ± 3.21%, which confers excellent flow properties, while the compressibility index of EF-4 was 14.81% ± 3.43%, suggesting a good flow property. Other three formulations showed fair flow properties (Table 4). In Hausner’s ratio, EF-2 provided excellent flow properties (1.02 ± 0.87), while EF-4 exhibited good properties (1.17 ± 0.59).

### 3.3. FT-IR Spectroscopy

**Figure 1:**
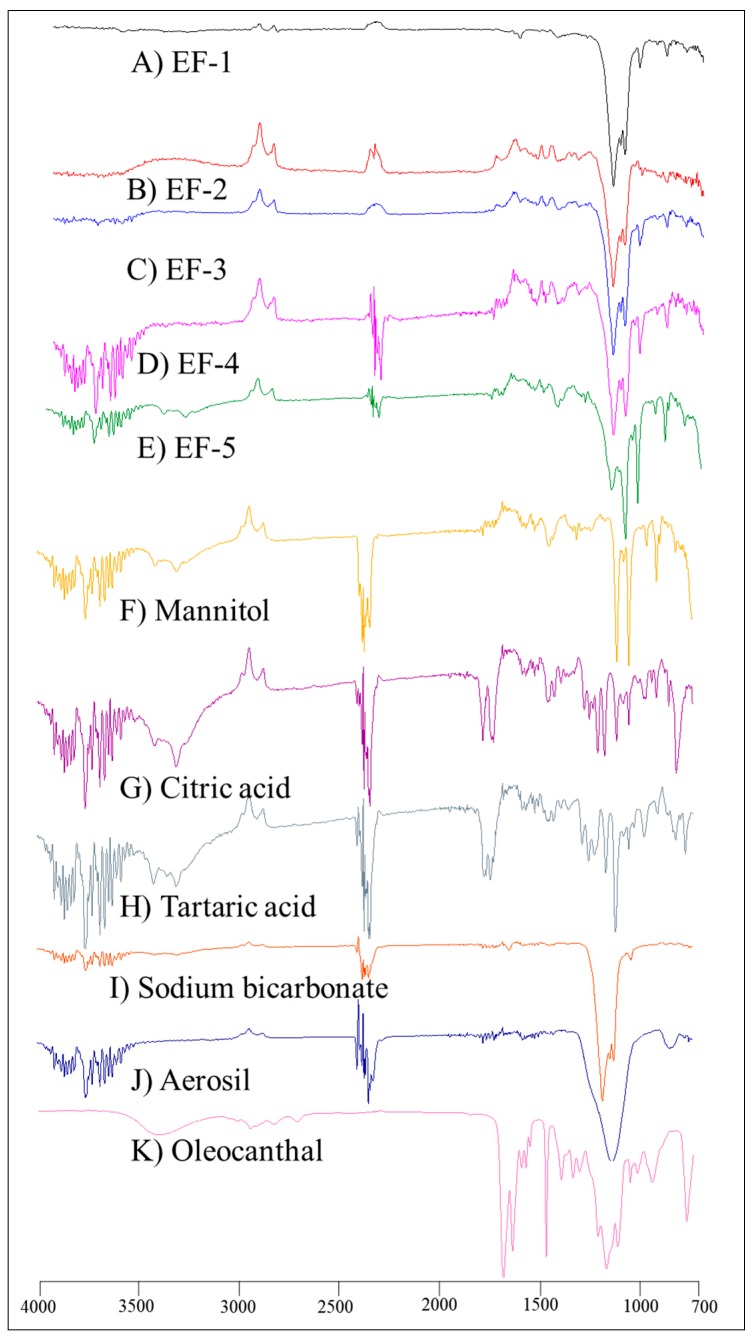
FT-IR spectra of (**A**) EF-1, (**B**) EF-2, (**C**) EF-3, (**D**) EF-4, **E**) EF-5, (**F**) mannitol, (**G**) citric acid, (**H**) tartaric acid, (**I**) sodium bicarbonate, (**J**) aerosil, and (**K**) plain non-formulated OC.

Unlike the plain non-formulated OC, the IR spectra of EF-1-EF-5 (Figure 1) showed significantly suppressed OC characteristic bands, possibly due to plausible entrapment of OC within the formulation carriers.

### 3.4. Differential Scanning Calorimetry (DSC)

**Figure 2:**
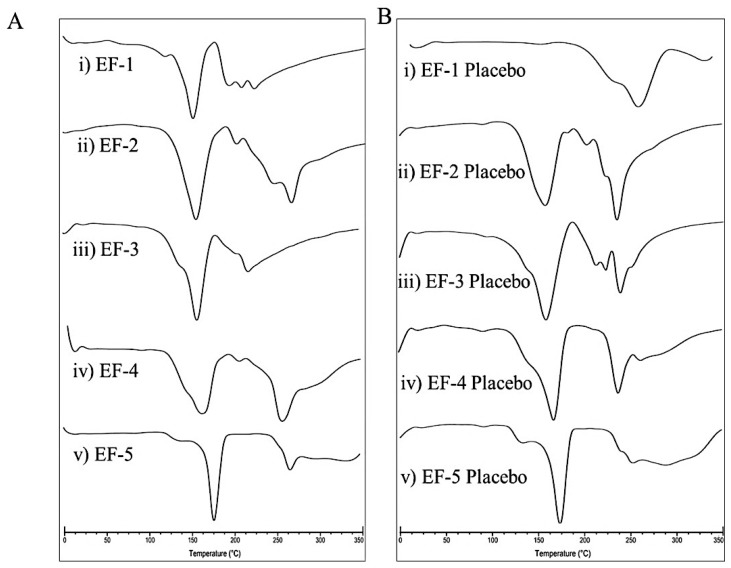
DSC thermograms of (**A**) (i) EF-1, (ii) EF-2, (iii) EF-3, (iv) EF-4, (v) EF-5, and (**B**) (i) EF-1 Placebo, (ii) EF-2 Placebo, (iii) EF-3 Placebo, (iv) EF-4 Placebo, (v) EF-5 Placebo.

DSC is a thermo-analytical technique that has been extensively used to confirm the alteration of physical state, phase transition, and decomposition of pharmaceuticals [35]. The DSC thermograms of the five effervescent powder formulations EF-1-EF-5 and their corresponding placebos are shown in Figure 2. The melting points of EF-1-EF-5 were 151.50, 152, 157.50, 162.50, and 175 °C, respectively. Meanwhile, the melting points of the formulation placebos were 161, 156, 157, 164, and 173.50 °C, respectively. Since OC is an oily liquid at room temperature, it is not possible to assess its melting point. Thus, it was difficult to predict the OC entrapment in the effervescent formulations. However, EF-1-EF-5 and their placebos have clearly different thermograms/endothermic peaks. Specifically, the sharp peaks with high intensities found in EF-1, EF-2, and EF-4 thermograms indicate successful OC entrapment within these formulations (Figure 2).

Based on favorable pH, effervescent time, flow properties, and other physical characteristics, EF-2 has been selected for subsequent studies.

### 3.5. Characterization of EF-2 Surface Morphology Using SEM and CLSM

**Figure 3:**
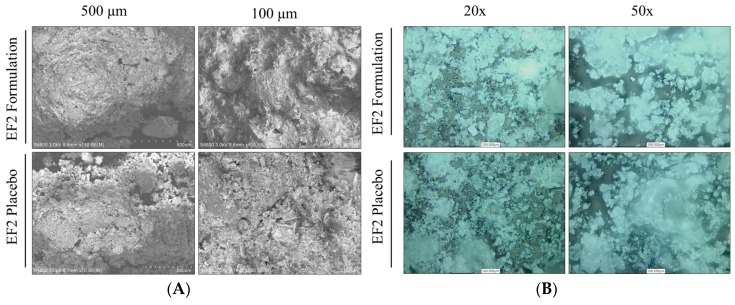
Microscopic characterization of EF-2. (**A**) Scanning electron micrographs of EF-2 and its placebo. (**B**) Confocal lesser scanning micrograph of EF-2 and its placebo.

The micro and macrostructural surface morphology of the EF-2 formulation and its placebo were investigated by using scanning electron microscopy (SEM) and confocal lesser scanning microscopy (CLSM). EF-2 formulation exhibited a more cohesive arrangement as compared to its placebo in SEM micrographs (Figure 3A). The placebo appeared as sub-angular–sub-rounded particles with a smooth surface with fine particles and wide gaps. Meanwhile, particles of OC were attached to the outer surface of the EF-2 powder and agglomerated forming clumps with a smooth surface. EF-2 formulation also has considerably uniform and smaller particle size as compared to its placebo formulation. While in CLSM, micrographs of EF-2 formulation exhibited more homogenous rectangular shape cubes with smooth surfaces, while the placebo exhibited irregular rough surface amorphous powder clumps (Figure 3B).

### 3.6. Dissolution Study

**Figure 4:**
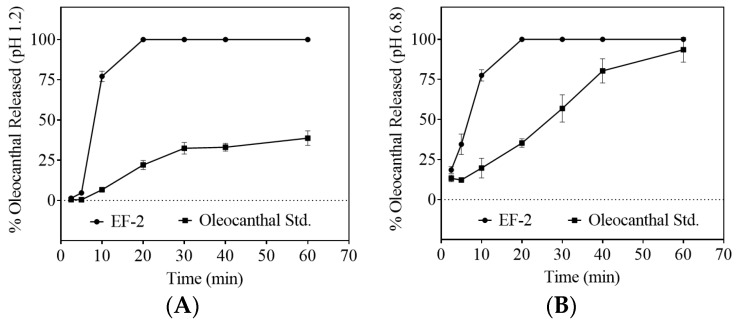
Comparison of the dissolution profile of plain non-formulated OC with OC in EF-2. (**A**) Dissolution profile in simulated gastric fluid (pH 1.2) without enzymes, and (**B**) dissolution profile in simulated intestinal fluid (pH 6.8) without enzymes.

Comparison of the dissolution profiles of the plain non-formulated OC with EF-2 formulation in artificial simulated gastric fluid (SGF, pH 1.2) and simulated intestinal fluid (SIF, pH 6.8) proved significant OC dissolution enhancement in EF-2 (Figure 4A,B, respectively). In SGF, within 10 min of the experiment, approximately 75% of OC in EF-2 was released and dissolved, whereas only 10% of plain OC was dissolved at this time point (Figure 4A). After 20 min, OC in EF-2 dissolved almost completely, unlike plain OC, which showed less than 25% dissolution (Figure 4A). The maximum plain OC dissolution was 40% after 60 min, which clearly highlights improved dissolution of OC in EF-2. Similarly, plain OC dissolved only 20%, while EF-2 provided 75% OC dissolution within 10 min in SIF. It took 20 min for 100% dissolution of OC in EF-2 in SIF, while plain OC exhibited 40% dissolution only (Figure 4B). This clearly shows the superior dissolution advantage for OC in EF-2 at both low and high pH conditions.

### 3.7. Taste Masking Evaluation of EF-2 Using the E-Tongue Taste Map and Taste Comparison

**Figure 5:**
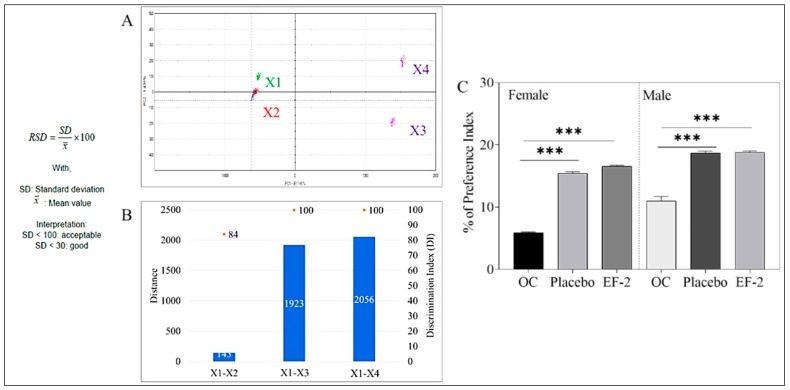
Assessment of OC taste masking in EF-2. (**A**) alpha MOS e-tongue generated taste map based on principal component analysis (PCA) of oleocanthal formulations. X1 = artificial saliva without enzyme; X2 = artificial saliva + plain non-formulated OC, X3 = EF-2; X4 = EF-2 placebo without OC; (**B**) distance between samples X1–X4; (**C**) animal preference test. Percentage of animal preference index of plain non-formulated OC, EF-2 formulation, and its placebo in female and male Swiss albino mice.

The comparative percentage between two principal component analyses (PCA1-PCA2) were used to describe the total variation of the data by converting the data acquired by the Alpha MOS e-tongue system using seven different taste sensors to two-dimensional data creating a taste map [29,30,36,37]. The system was able to differentiate between the taste map analysis of the artificial saliva vehicle control (X1) and the plain non-formulated OC (X2, Figure 5). EF-2 formulation solution (X3) was compared with its placebo (X4). The Euclidian distances between X3 and X4 were calculated to assess the taste proximity between both samples. The lower the distance, the closer the sensed taste. A Discrimination Index (DI in %) was determined for EF-2 and its placebo. This indicator accounts for the average difference between the pairs to compare, as well as the dispersion of each sample. The closer the index to 100%, the greater the distance between the centers of gravity and the smaller the dispersion within groups. Thus, DI assesses the significance of taste difference between the groups. X1–X2 distance was very small in comparison to the other pair, X3 and X4 distance (Figure 5A). Meanwhile, X3 and X4 were the most distant samples compared to all samples, which clearly indicated effective taste masking of the original plain OC (X2) irritative taste versus OC taste in the formulation EF-2 (Figure 5A). The DI for EF-2 formulation and its placebo was 84% and 90%, respectively, while the plain non-formulated OC (X2) DI was less than 10% (Figure 5B). The higher DI values demonstrated significant taste-masking between X2 versus X3 and X4. The taste analysis of OC in EF-2 formulation using the Alpha MOS e-tongue proved formulation EF-2 (X3) and its placebo (X4) are the most distinct samples due to effective taste masking possible imparted by carbonic acid (Figure 5B).

### 3.8. Animal Preference Test

EF-2 formulation taste preference was assessed in vivo using a Swiss albino mouse model. Animals had access to bottles containing either plain non-formulated OC, EF-2, or its placebo effervescent formulation. At the end of the two-bottle two-day animal preference experiment, the preference index of plain non-formulated OC was 5.88% and 11.0% in female and male mice, respectively (Figure 5C). On the other hand, EF-2 formulation preference index was 15.42% and 18.66% in female and male mice, respectively (Figure 5C). The preference index for the placebo formulation was very close to EF-2 but much higher than the plain non-formulated OC, confirming effective taste masking (Figure 5C).

### 3.9. Evaluation of EF-2 Formulation Effects In Vitro Against BC Cells

**Figure 6:**
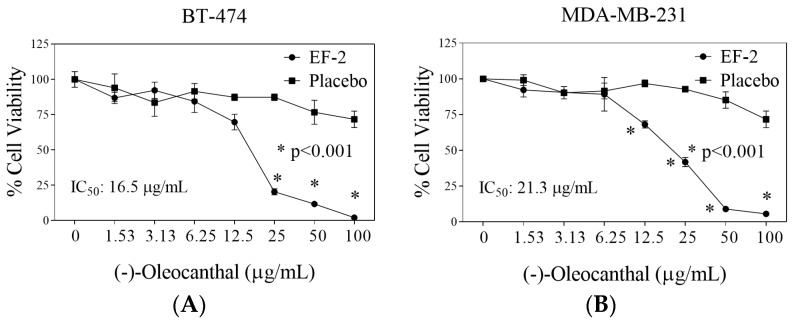
Comparison of the in vitro effects of EF-2 and its placebo on the growth of ER^+^/HER2^+^ BC cells BT-474 and the TNBC MDA-MB-231 cells. (**A**) Effect of EF-2 and its placebo treatment on the growth of BT-474 cells after 48 h culture period. (**B**) Effect of EF-2 and its placebo treatment on the growth of MDA-MB-231 cells after the 48 h culture period. Cells were plated at a density of 1×10^4^ cells/well in 96-well plates and maintained in media supplemented with 10% FBS and allowed to adhere overnight. Cells were then treated with placebo or increasing EF-2 concentrations in serum-free media for 48 h. At the end of treatment, the viable cell number was determined by the MTT colorimetric assay. Vertical bars indicate mean cell count ± SD (*n* = 6) in each treatment group. *p < 0.05 as compared with vehicle-treated controls.

The in vitro effects of EF-2 and its placebo were evaluated against the growth and viability of two BC cell lines using MTT assay (Figure 6). The triple-negative breast cancer (TNBC) cells MDA-MB-231 do not express estrogen receptor (ERα) nor HER2, but expresses c-MET, which is considered the main OC molecular target. Dysregulated c-MET in TNBC correlates with survival and aggressive motility pattern [7,19,23,38]. On the other hand, the BT-474 BC cells express ERα, HER2, and c-MET expression in lesser extent compared to TNBC cells [7,23,38]. The antiproliferative activity of different OC doses in EF-2 and its placebo was evaluated against MDA-MB-231 and BT-474 BC cells after 48 h treatment period (Figure 6). The IC_50_ values of EF-2 were 21.3 and 16.5 µg/mL against MDA-MB-231 and BT-474 cells, respectively (Figure 6). It is worth noting that the modest OC in vitro activity does not match its potent in vivo activity, which may suggest possible metabolic bioactivation [7,23,26,33,39].

### 3.10. Evaluation of In Vivo Oral Early EF-2 Treatment Activity Against the Growth of ER^+^/HER2^+^ BC in Nude Mouse Xenograft Model

**Figure 7:**
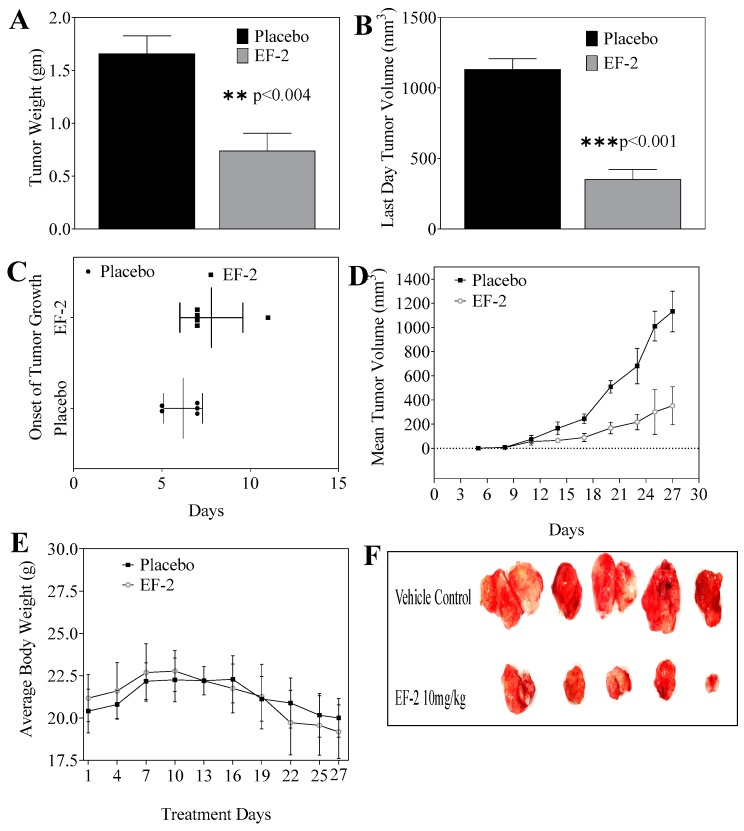
Comparison of the in vivo effects of EF-2 and its placebo on the progression of BT-474 BC cells in the athymic nude mouse orthotopic xenograft model. (**A**) Vertical bars represent mean tumor weight at the end of the experiment. (**B**) Vertical bars represent mean tumor volume at the end of the experiment. (**C**) Comparison of the onset of tumor initiation in animals treated with EF-2 and its placebo. (**D**) Monitoring the tumor volume in different treatments over the experiment course. Points represent the mean tumor volume of several tumors (*n* = 5) in each experimental group during the treatment period. Error bars indicate SD for *n* = 5. (**E**) Monitoring mice body weight over the experiment course. (**F**) Excised primary tumors of each experimental group after the end of the tumor growth experiment. Top row: mice tumors obtained from the placebo-treated control group. Bottom row: tumors excised from mice treated with OC 10 mg/kg in EF-2 formulation.

The in vivo antitumor oral efficacy of early EF-2 treatment was assessed using an orthotopic xenograft mouse model of BT-474 cells in nude mice. At the end of the experiment, the mean tumor weight of mice treated with EF-2 and its placebo were 0.74 ± 0.36 g and 1.66 ± 0.37 g, respectively (Figure 7A). Furthermore, the mean tumor volume was 352.23 ± 156.88 mm^3^ and 1132.38 ± 168.39 mm^3^ for EF-2 formulation and placebo-treated mice groups, respectively (Figure 7B). The average onset of tumor development was six days in placebo control mice and eight days in EF-2 treated animals (Figure 7C). A daily oral 10 mg/kg OC dose in EF-2 resulted 70% tumor growth reduction in comparison with the placebo-treated control animals (Figure 7D,F). EF-2 treatment did not cause any significant animal weight change over the experiment course, compared to the placebo treatment (Figure 7E).

### 3.11. Evaluation of In Vivo Oral EF-2 Ability to Inhibit the ER^+^/HER2^+^ BC Locoregional Recurrence in Nude Mouse Xenograft Model After Primary Tumor Surgical Excision

**Figure 8:**
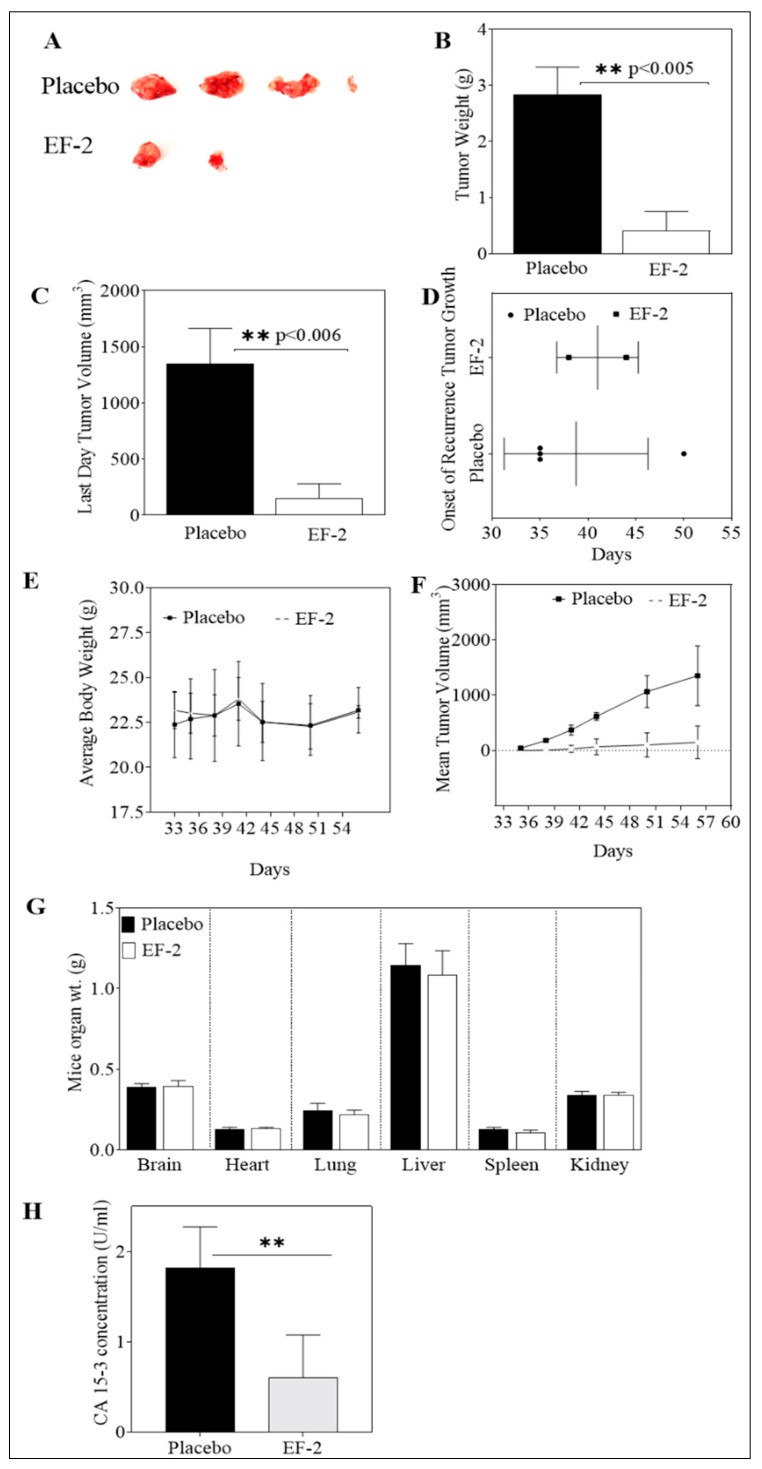
Effect of daily OC 10 mg/kg oral treatment in EF-2 on the recurrence of BT-474 BC cells after primary tumor surgical excision in the nude mouse xenograft model. (**A**) Recurred mice tumors at the experiment end. Top row: recurred mice tumors of the placebo-treated group. Bottom row: recurred mice tumors of EF-2-treated group. (**B**) Vertical bars comparing the mean recurrence tumor weight at the end of the experiment. (**C**) Vertical bars representing the mean recurrence tumor volume at the end of the experiment. (**D**) Comparison of effects of EF-2 and placebo treatments on the recurrence tumor onset. (**E**) Body weight monitoring of animals over the duration of the experiment. (**F**) Recurrence tumor volumes monitoring over the experiment course. Points represent the mean tumor volume of recurred tumors (*n* = 5) in each experimental group during the treatment period. Error bars indicate SD for *n* = 5. (**G**) Comparison of the effects of EF-2 and placebo treatments on the animal organ weights at the end of the experiment. (**H**) Comparison of the effects of EF-2 and placebo treatments on the serum levels of the recurrence tumor biomarker CA 15-3 at the end of the experiment.

Mice in previous tumor growth experiments were subjected to primary tumor surgical resection on experiment day 27. These mice were then used to assess the ability of EF-2 to inhibit the human HER2^+^/ER^+^ BC BT-474 cells locoregional recurrence. Daily oral OC 10 mg/kg treatments in EF-2 continued the next day after surgery until experiment day 57. The mean weight of recurrence tumors in EF-2 and its placebo-treated was 0.41 ± 0.70 g and 2.83 ± 0.84 g, respectively, at the end of the experiment. Furthermore, the mean recurrence tumor volume was 148.17 ± 297.62 mm^3^ and 1351 ± 542.97 mm^3^ for EF-2 and placebo-treated mice groups, respectively (Figure 8A–C,F). A higher SD was observed in the EF-2 treatment group since only two out of five mice developed recurrence tumors, unlike the placebo-treated group in which four out of five mice developed recurrence tumors. EF-2 formulation treatment exhibited about 90% tumor recurrence reduction compared to the placebo-treated animals. The average onset of tumor recurrence was 42 and 38 days in EF-2 and its placebo control-treated mice (Figure 8D). On the other hand, mice body weights, clinical, and physical behavior were monitored throughout the study course and showed no observed significant changes (Figure 8E). Furthermore, different collected organs after mice were sacrificed at the end of the experiment showed no significant gross weight alterations between the EF-2 and placebo-treated animals (Figure 8G).

### 3.12. In Vivo Effects of EF-2 Treatment on the Level of the Human Serum CA 15-3 Biomarker in HER2^+^/ER^+^ BC Recurrence Model

The level of the cancer-associated antigen CA 15-3 was measured in collected recurrence experiment mice serum samples of both EF-2 and placebo-treated groups (Figure 8H). In placebo-treated group mice, the mean level of CA 15-3 in sera was 1.82 ± 0.45 U/mL whereas, the mean level of CA 15-3 was 0.60 ± 0.31 U/mL in sera collected from EF-2 treated group mice (Figure 8H). This finding highlights the potential of OC treatment in EF-2 to minimize the ER^+^/HER2^+^ BC recurrence risk.

### 3.13. Western Blotting Analysis

**Figure 9:**
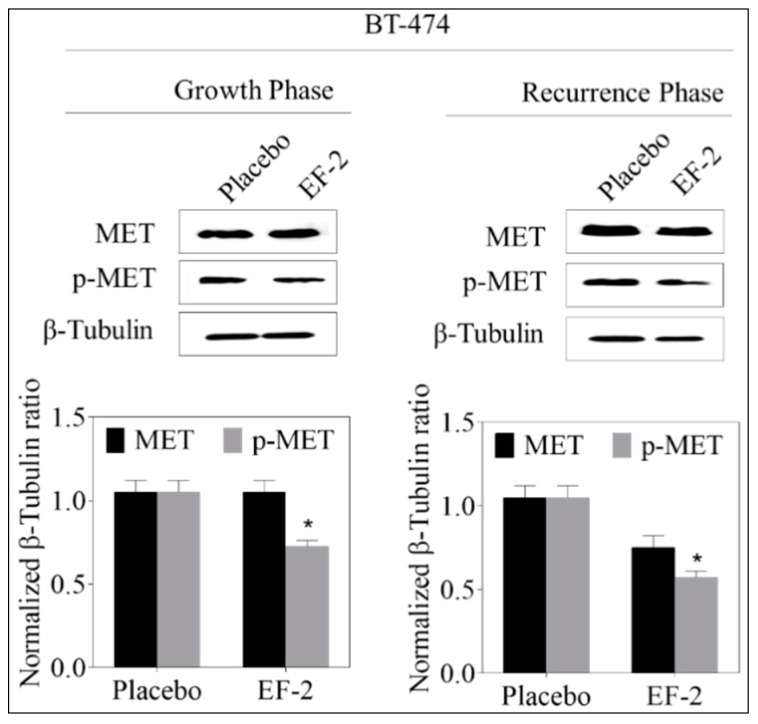
Evaluation of the pharmacodynamic effects of EF-2 via assessing its suppressive effect on c-MET activation, the main OC validated molecular target in BT-474 cells in nude mouse xenograft models. Left panel: Western blot analysis of excised primary tumors. Right panel: western blot analysis of recurrence tumors. Bottom panels: Scanning densitometric analysis and the integrated optical density of each band was normalized with corresponding β-tubulin. Error bars indicate ± SEM for *n* = 5. *p < 0.05 as compared to vehicle-treated controls.

**Figure 10:**
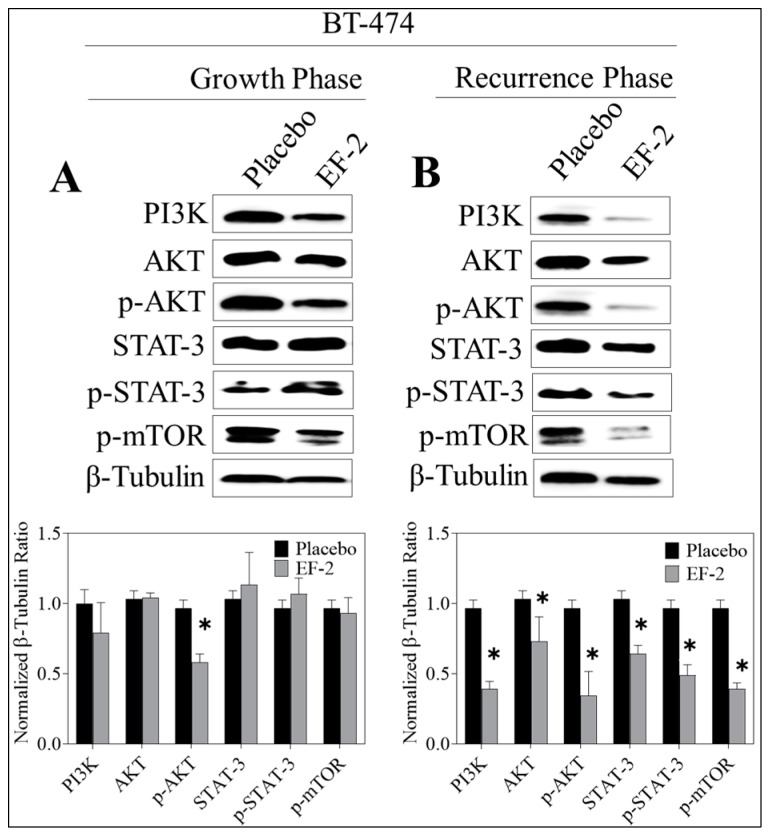
Comparison of the c-MET downstream signaling proteins in BT-474 tumor cell lysates of primary excised tumors (early treatment phase) and recurrence tumors (recurrence phase) in response to treatment with EF-2 and its placebo control in nude mouse xenograft models. (**A**) Upper left panel: Western blot analysis of excised primary tumors. Lower left panel: densitometric quantification of the Western blotting bands. (**B**) Upper right panel: Western blotting analysis of recurrence tumor downstream markers. Lower right panel: densitometric quantification of the Western blot bands. Scanning densitometric analysis and the integrated optical density of each band was normalized with corresponding β-tubulin. Error bars indicate ± SEM for *n* = 5. *p < 0.05 as compared with vehicle-treated controls.

Western blot analysis of the excised primary tumors showed a 35% reduction of activated c-MET (Y1234/1235) level in EF-2 treated mice when compared to the placebo-treated control group animals without any change of the total c-MET level. Meanwhile, the western blotting examination of the recurred tumors collected after animals sacrificed showed a marked reduction (47%) in the intra-tumoral level of activated c-MET and about a 28% reduction in the total c-MET levels in EF-2 treated mice tumors compared to the placebo-treated control group tumors (Figure 9). Comparative investigation of the effects of EF-2 and its placebo treatments on downstream signaling markers in primary excised and recurrence tumor cell lysates of nude mouse models showed significant downregulation of several markers. In early treatment mode, p-Akt was significantly downregulated by EF-2 treatment, along with a slight reduction in levels of PI3K and p-mTOR and a slight increase in the total and p-STAT3 levels. However, more importantly, EF-2 treatment showed significant inhibition of PI3K and activation of AKT, STAT-3, and mTOR levels, compared to the placebo-treated group in recurred tumors, which are known to be more aggressive and resistant versus its parent primary tumors (Figure 10).

## 4. Discussion

(–)-Oleocanthal is an exceptional natural phenolic compound exclusively occurring in EVOO with documented activities against cancer, inflammation, and Alzheimer’s disease [1]. OC causes oropharynx irritative sensation via the activation of the transient receptor potential cation channel subfamily A, member 1 (TRPA1) receptor [12,13]. Despite its extensive biological studies, there is no OC systemic formulation reported so far to be used for subsequent preclinical and clinical applications. Thus, a taste-masked OC formulation is required to take this unique bioactive natural product to the next level, including future clinical assessments and therapeutic applications as a prospective nutraceutical. The oropharynx irritative taste of OC is not generalized acid-sensing and did not correlate with acid-induced irritation [13]. OC and carbonic acid taste intensity ratings and mechanisms proved independent and were not correlated, which justify effective taste masking by the use of effervescent formulations [13]. On the other hand, effervescent formulations have gained popularity in over–the–counter supplemental and pharmaceutical applications due to their easiness of use and favorable taste. The effervescent technique can also accelerate drug disintegration and dissolution, especially in quick-release preparations [26].

This study optimized an OC effervescent powder formulation using various ratios of citric and tartaric acids and sodium bicarbonate. The dissolution of OC in water was significantly enhanced by reducing the solution pH due to the CO_2_ release and formation of carbonic acid. The dissolution studies conducted at different physiologic pH mimicking the gastric and intestinal fluids suggested better palatability and improved absorption rate. The acidic pH of the solution was also important for improved formulation taste, whereas the acidic solution exhibits better palatability and good oral absorption [26]. The measured pH of all formulations was below 6, which is considered an acceptable pH range [26].

Favorable taste of oral formulation plays a prime role for ensuring patient acceptability, compliance, and commercial success. The taste assessment of EF-2 was essential to determine the success level for OC irritative taste masking. The e-tongue is an array of nonspecific, low-selectivity, chemical sensors with high stability and cross-sensitivity to different species in solution [29,30]. The e-tongue effectively discriminated the taste sensing of plain non-formulated OC on one side and the OC in EF-2 OC, and EF-2 placebo without OC on the other side. EF-2 and its placebo were not discriminated from each other as they nearly had similar DI values, indicating successful OC taste masking in EF-2. Effective taste masking was further confirmed by the animal preference test in which Swiss albino mice differentiated between plain non-formulated OC and EF-2, as the later was significantly favored and frequently consumed unlike the former, which had an unmasked irritative taste. Meanwhile, mice could not discriminate between EF-2 and its placebo formulation, as both had nearly similar preference. 

Unlike its modest in vitro activity, extensive in vivo anticancer studies of OC have documented the remarkable tumor xenograft growth inhibitory potency not only against diverse molecular phenotypes of BC but also against a wide array of diverse tumors [1,3,7]. Recent literature evidenced the superior OC activity against the luminal B BC represented by the ER^+^/HER^+^ BT-474 cells [23]. This particular BC phenotype sensitivity is attributed to its overexpression of both OC validated molecular targets c-MET and ERα [7,19,23]. This study confirmed the comparable in vivo potency of OC in EF-2 versus plain non-formulated OC against BT-474 BC progression using identical experiment conditions [23,33]. This study used an early treatment mode by starting EF-2 treatments immediately the second day after tumor cells xenografting based on earlier studies showing improved OC efficacy in this mode [7,23,26]. It is, therefore, expected that OC in EF-2 was affecting the viability and colonization ability of engrafted tumor cells before solid tumor formation, which ultimately could contribute to the obtained delayed tumor growth. Recurrence of BC is one of the main mortality reasons among patient survivors who completed therapeutic interventions [40]. To date, there are no formal recurrence inhibitors, as dormant tumor cells causing recurrence are usually resistant to existing targeted, chemo, and radio therapies [33]. In addition, numerous studies suggested that different molecular subtypes of BC have a differential recurrence pattern [41]. Clinical evidence demonstrates that TNBC is associated with higher five-year recurrence risk versus the 10-year recurrence range for the ER^+^/HER2^+^ BC [40]. In this study, daily oral 10 mg/kg OC in EF-2 taken in adjuvant mode (post-surgical excision of the primary tumor) effectively inhibited BT-474 BC cells locoregional recurrence in nude mice xenograft after primary tumor surgical excision [33]. It is expected that larger primary tumors are more likely to develop recurrence tumors after primary tumor resection compared to smaller tumors [33,42,43,44,45,46]. However, this study was designed to use the same animals to assess the OC in EF-2 suppressive efficacy for tumor growth and recurrence, mimicking clinical situations in which patients can use both neoadjuvant and adjuvant therapies or interventions before and after surgical tumor resection, respectively. Therefore, the final adjuvant mode suppressive effect of OC in EF-2 on tumor recurrence in this study also correlates well with its neoadjuvant mode suppression of the primary tumor. This finding further highlights the unique translational potential of EF-2 as a nutraceutical formulation of OC for long-term use to prevent luminal breast tumors recurrence supported by the safe food consumption of EVOO over human history.

The C-1 and C-3 aldehyde functionalities are associated with extensive unpredictable reactivity with endogenous amino acids, peptides, and proteins, and therefore, the pharmacokinetics of OC have never been identified. Extensive studies have validated the c-MET RTK and its downstream pathways including mTOR, Akt, STAT3, ERK1/2, MAPK, and HSP90 as OC in vivo anti-BC molecular targets [7,9,18,23,26,33,39]. c-MET dysregulation can trigger aggressive proliferation and activation mechanisms of quiescent or dormant tumor cells, repopulation, and subsequent relapse and recurrence in breast and several other cancers [42,43,44,45,46]. On the other hand, amplification of c-MET was also associated with the escape of cancer cells from the anticancer effects of several targeted therapies [7,26,33,43]. Therefore, this study relied on assessing the pharmacodynamics of OC in EF-2 by quantifying and comparing the activated and total c-MET levels in EF-2-treated and placebo-treated control tumor cell lysates. EF-2 treatment showed good suppression of p-c-MET levels in tumor progression mode and impressively reduced both the total and activated c-Met levels in recurrence tumors. Interestingly, EF-2 treatment significantly downregulated PI3K levels and suppressed the activation of p-AKT, p-STAT-3, and p-mTOR in recurrence tumors compared to the placebo group. Recurrence tumors are known to be more aggressive and invasive compared to their primary tumors, further confirming the recurrence suppressive potential of OC treatment [33,40,41,44]. Suppression of activated MET and downstream effectors in tumors further validated the pharmacodynamics efficacy of OC in EF-2.

It is well documented that each 2.6 adult mouse days are equivalent to one human year [47]. After xenografting, treatment was started immediately to observe whether EF-2 exhibited any tumor progression delay. Here, EF-2 delayed the tumor progression onset by two days and tumor recurrence onset by four days. Therefore, OC treatment in EF-2 is predicted to delay luminal B breast tumor development and locoregional recurrence in humans by 9.2 and 18.5 months, respectively. In addition, the effect of EF-2 on the onset of tumor progression may be a reflection of the alteration of tumor engraftment or cell viability prior to engraftment as alternative mechanisms for obtaining the observation of the delayed growth curve.

The cancer antigen 15-3 (CA 15-3) is a protein produced by a variety of cells, especially BC cells and used as a tumor recurrence biomarker. CA 15-3 levels are higher than normal in most women with advanced metastatic BC [33]. More than 96% of BC patients with local and systemic recurrence have elevated levels of CA 15-3 [33]. EF-2-treated mice showed significantly reduced CA 15-3 sera levels compared to the placebo, indicating the potential of OC formulated in EF-2 as plausible BC recurrence inhibitor.

## 5. Conclusions

The daily high OC consumption through its dietary source is common, especially in Mediterranean populations consuming EVOO. OC is a bioactive micronutrient in a key Mediterranean diet ingredient (EVOO), which may correlate with positive findings in several epidemiological studies suggestive of less incidence of breast, colon, and other malignancies compared to western and other populations. The current study has been progressively building upon previous well-developed reports, which highlighted OC as a unique c-MET kinase inhibitor for the control of BC progression and locoregional recurrence. Effervescent formulation EF-2 successfully tackled the OC oropharynx irritative taste challenge and showed an impressive taste-masking profile validated by both in vitro and in vivo models. EF-2 enhanced OC dissolution and release, which translated to effective in vivo efficacy in BC progression and locoregional recurrence models. OC EF-2 formulation represents a prospective nutraceutical/dietary supplement appropriate for use by patients, survivors, and women at risk of disease to control and prevent BC progression and recurrence.

## Figures and Tables

**Table 1:** Optimization of different effervescent ingredients.

Code	Citric Acid (mg)	Tartaric Acid (mg)	Sodium Bicarbonate (mg)	Effervescence Time (sec)	pH
P1	84.0	166.0	250.0	54.00 ± 0.31	5.63 ± 0.23
P2	80.0	160.0	240.0	55.00 ± 0.46	5.61 ± 0.11
P3	74.0	146.0	220.0	46.00 ± 0.38	5.60 ± 0.21
P4	74.0	160.0	220.0	42.00 ± 0.21	5.67 ± 0.35
P5	40.0	80.0	120.0	51.00 ± 0.51	5.23 ± 0.24
P6	120.0	0.0	80.0	43.00 ± 0.53	4.48 ± 0.32
P7	120.0	0.0	90.0	45.00 ± 0.42	4.43 ± 0.21
P8	120.0	0.0	120.0	46.00 ± 0.34	4.53 ± 0.23
P9	0.0	120.0	120.0	30.00 ± 0.22	4.42 ± 0.43
P10	0.0	250.0	250.0	28.00 ± 0.37	4.27 ± 0.26
P11	84.0	84.0	250.0	54.00 ± 0.71	4.42 ± 0.27
P12	84.0	126.0	250.0	50.00 ± 0.11	4.39 ± 0.31
P13	84.0	252.0	250.0	37.00 ± 0.41	4.27 ± 0.26
P14	84.0	336.0	250.0	24.00 ± 0.45	4.25 ± 0.35
P15	166.0	166.0	250.0	28.00 ± 0.36	4.31 ± 0.21
P16	42.0	84.0	250.0	55.00 ± 0.43	4.58 ± 0.41
P17	63.2	84.0	250.0	43.00 ± 0.53	4.52 ± 0.32
P18	126.0	84.0	250.0	31.00 ± 0.45	4.40 ± 0.49
P19	168.0	84.0	250.0	54.00 ± 0.32	4.32 ± 0.25
P20	0.0	84.0	250.0	54.00 ± 0.41	4.52 ± 0.27
P21	84.0	0.0	250.0	42.00 ± 0.44	4.75 ± 0.32
P22	168.0	0.0	250.	36.00 ± 0.42	4.49 ± 0.30
P23	126.0	0.0	250.0	58.00 ± 0.18	4.59 ± 0.28
P24	126.0	0.0	125.0	78.00 ± 0.39	4.58 ± 0.41
P25	126.0	0.0	187.5	48.00 ± 0.52	4.54 ± 0.29
P26	126.0	0.0	375.0	55.00 ± 0.22	4.55 ± 0.22
P27	126.0	0.0	500.0	39.00 ± 0.37	4.52 ± 0.31

**Table 2:** Composition of optimized effervescent formulations EF-1-EF-5.

Ingredients (mg)	Formulation Code				
	EF-1	EF-2	EF-3	EF-4	EF-5
Oleocanthal	10	10	10	10	10
Citric acid	84	80	74	40	120
Tartaric acid	166	160	146	80	-
Sodium bicarbonate	250	240	220	160	80
Aerosil-200	20	20	20	20	20
Mannitol	460	480	440	680	760
Flavor	10	10	10	10	10

**Table 3:** Physicochemical evaluation of different effervescent powder formulations.

Formulation Code	CO_2_ Content (mg)	pH	Effervescence Time (sec)
EF-1	44.33 ± 7.77	3.86 ± 0.32	42.67 ± 1.41
EF-2	63.00 ± 2.00	4.36 ± 0.11	68.00 ± 3.78
EF-3	37.67 ± 9.87	3.21 ± 0.20	47.00 ± 2.12
EF-4	59.67 ± 4.04	3.68 ± 0.41	40.00 ± 5.66
EF-5	59.67 ± 18.56	3.95 ± 0.17	36.67 ± 5.66

**Table 4:** Flow properties of effervescent powder formulations.

Formulation Code	Bulk Density	Tapped Density	Hausner’s Ratio	Angle of Repose (*θ*) (Degrees)	Carr’s Index
EF-1	0.50 ± 0.11	0.60 ± 0.02	1.20 ± 0.58	33.82 ± 2.87	16.67 ± 4.32%
EF-2	0.58 ± 0.15	0.60 ± 0.23	1.02 ± 0.87	28.68 ± 3.14	5.00 ± 3.21%
EF-3	0.46 ± 0.21	0.58 ± 0.21	1.25 ± 0.92	25.64 ± 1.87	19.97 ± 4.54%
EF-4	0.54 ± 0.17	0.46 ± 0.31	1.17 ± 0.59	33.82 ± 3.01	14.81 ± 3.43%
EF-5	0.39 ± 0.13	0.48 ± 0.22	1.23 ± 0.62	20.80 ± 2.43	18.75 ± 3.33%

## References

[1] Pang K.L., Chin K.Y. (2018). The biological activities of oleocanthal from a molecular perspective. Nutrients.

[2] Parkinson L., Keast R. (2014). Oleocanthal, a phenolic derived from virgin olive oil: A review of the beneficial effects on inflammatory disease. Int. J. Mol. Sci..

[3] Cicerale S., Lucas L., Keast R. (2010). Biological activities of phenolic compounds present in virgin olive oil. Int. J. Mol. Sci..

[4] Cicerale S., Lucas L.J., Keast R.S. (2012). Antimicrobial, antioxidant and anti-inflammatory phenolic activities in extra virgin olive oil. Curr. Opin. Biotechnol..

[5] Segura-Carretero A., Curiel J.A. (2018). Current disease-targets for oleocanthal as promising natural therapeutic agent. Int. J. Mol. Sci..

[6] Andrewes P., Busch J.L., de Joode T., Groenewegen A., Alexandre H. (2003). Sensory properties of virgin olive oil polyphenols: Identification of deacetoxy-ligstroside aglycon as a key contributor to pungency. J. Agric Food Chem..

[7] Akl M.R., Ayoub N.M., Mohyeldin M.M., Busnena B.A., Foudah A.I., Liu Y.Y., Sayed K.A. (2014). Olive phenolics as c-Met inhibitors: (-)-Oleocanthal attenuates cell proliferation, invasiveness, and tumor growth in breast cancer models. PLoS ONE.

[8] Montedoro G., Maurizio M., Baldioli M., Miniati E. (1992). Simple and hydrolyzable phenolic compounds in virgin olive oil. 1. Their extraction, separation, and quantitative and semiquantitative evaluation by HPLC. J. Agric. Food Chem..

[9] Montedoro G., Servili M., Baldioli M., Selvaggini R., Miniati E., Macchioni A. (1993). Simple and hydrolyzable compounds in virgin olive oil. 3. Spectroscopic characterizations of the secoiridoid derivatives. J. Agric. Food Chem..

[10] Beauchamp G.K., Keast R.S., Morel D., Lin J., Pika J., Han Q., Lee C.H., Smith A.B., Breslin P.A. (2005). Phytochemistry: Ibuprofen-like activity in extra-virgin olive oil. Nature.

[11] Scotece M., Gómez R., Conde J., Lopez V., Gómez-Reino J.J., Lago F., Smith A.B., Gualillo O. (2012). Further evidence for the anti-inflammatory activity of oleocanthal: Inhibition of MIP-1α and IL-6 in J774 macrophages and in ATDC5 chondrocytes. Life Sci..

[12] Peyrot des Gachons C., Uchida K., Bryant B., Shima A., Sperry J.B., Dankulich-Nagrudny L., Tominaga M., Smith A.B., Beauchamp G.K., Breslin P.A. (2011). Unusual pungency from extra-virgin olive oil is attributable to restricted spatial expression of the receptor of oleocanthal. J. Neurosci..

[13] Cicerale S., Breslin P.A.S., Beauchamp G.K., Keast R.S.J. (2009). Sensory characterization of the irritant properties of oleocanthal, a natural anti-inflammatory agent in extra-virgin olive oils. Chem. Senses.

[14] Batarseh Y.S., Mohamed L.A., Al Rihani S.B., Mousa Y.M., Siddique A.B., El Sayed K.A., Kaddoumi A. (2017). Oleocanthal ameliorates amyloid-beta oligomers’ toxicity on astrocytes and neuronal cells: *In vitro* studies. Neuroscience.

[15] Qosa H., Batarseh Y.S., Mohyeldin M.M., El Sayed K.A., Keller J.N., Kaddoumi A. (2015). Oleocanthal enhances amyloid-beta clearance from the brains of TgSwDI mice and *in vitro* across a human blood-brain barrier model. ACS Chem. Neurosci..

[16] Monti M.C., Margarucci L., Riccio R., Casapullo A. (2012). Modulation of tau protein fibrillization by oleocanthal. J. Nat. Prod..

[17] Pitt J., Roth W., Lacor P., Smith A.B., Blankenship M., Velasco P., De Felice F., Breslin P., Klein W.L. (2009). Alzheimer’s-associated Abeta oligomers show altered structure; immunoreactivity and synaptotoxicity with low doses of oleocanthal. Toxicol. Appl. Pharmacol..

[18] Pei T., Meng Q., Han J., Sun H., Li L., Song R., Sun B., Pan S., Liang D., Liu L. (2016). (-)-Oleocanthal inhibits growth and metastasis by blocking activation of STAT3 in human hepatocellular carcinoma. Oncotarget.

[19] Elnagar A.Y., Sylvester P.W., El Sayed K.A. (2011). (-)-Oleocanthal as a c-Met inhibitor for the control of metastatic breast and prostate cancers. Planta Med..

[20] Subramaniam A., Shanmugam M.K., Perumal E., Li F., Nachiyappan A., Dai X., Swamy S.N., Ahn K.S., Kumar A.P., Tan B.K. (2013). Potential role of signal transducer and activator of transcription 3 (STAT3) signaling pathway in inflammation, survival, proliferation and invasion of hepatocellular carcinoma. Biochim Biophys. Acta..

[21] Polini B., Digiacomo M., Carpi S., Bertini S., Gado F., Saccomanni G., Macchia M., Nieri P., Manera C., Fogli S. (2018). Oleocanthal and oleacein contribute to the *in vitro* therapeutic potential of extra virgin oil-derived extracts in non-melanoma skin cancer. Toxicol. In Vitro..

[22] LeGendre O., Breslin P.A., Foster D.A. (2015). (-)-Oleocanthal rapidly and selectively induces cancer cell death via lysosomal membrane permeabilization. Mol. Cell Oncol..

[23] Ayoub N.M., Siddique A.B., Ebrahim H.Y., Mohyeldin M.M., El Sayed K.A. (2017). The olive oil phenolic (-)-oleocanthal modulates estrogen receptor expression in luminal breast cancer in vitro and in vivo and synergizes with tamoxifen treatment. Eur. J. Pharmacol..

[24] Padmanabhan B., Shetty R., Kulkarni V., Sen H., Bhushan I. (2014). Effervescent Composition and Method of Making it. WIPO (PCT).

[25] Parikh M.D. (2005). Effervescent powder. Hand Book of Pharmaceutical Granulation Technology.

[26] Siddique A.B., Ebrahim H.E., Qusa M., Btarsah Y., Fayaad A., Tajmim A., Nazzal S., Kaddoumi A., El Sayed K.A. (2019). Novel liquid-liquid extraction and self-emulsion methods for simplified isolation of extra-virgin olive oil phenolics with emphasis on (-)-oleocanthal and its oral anti-breast cancer activity. PLoS ONE.

[27] Jacob S., Shirwaikar A., Nair A. (2009). Preparation and evaluation of fast-disintegrating effervescent tablets of glibenclamide. Drug Dev. Ind. Pharm..

[28] Train D. (1958). Some aspects of the property of angle of repose of powders. J. Pharm. Pharmacol..

[29] Wei Y., Nedley M.P., Bhaduri S.B., Bredzinski X., Boddu S.H.S. (2015). Masking the bitter taste of injectable lidocaine HCl formulation for dental procedures. AAPS PharmSciTech.

[30] Podrazka M., Baczynska E., Kundys M., Jelen P.S., Nery E.W. (2018). Electronic tongue-A tool for all tastes?. Biosensors.

[31] Nesil T., Kanit L., Pogun S. (2005). Bitter taste and nicotine preference: Evidence for sex differences in rats. Am. J. Drug Alcohol Ab..

[32] Riss T.L., Moravec R.A., Niles A.L., Duellman S., Benink H.A., Worzella T.J., Minor L., Weidner J. (2004). Cell viability assays. Assay Guidance Manual.

[33] Siddique A.B., Ayoub N.M., Tajmim A., Meyer S.A., Hill R.A., El Sayed K.A. (2019). (-)-Oleocanthal prevents breast cancer locoregional recurrence after primary tumor surgical excision and neoadjuvant targeted therapy in orthotopic nude mouse models. Cancers.

[34] Aslani A., Fattahi F. (2013). Formulation, characterization and physicochemical evaluation of potassium citrate effervescent tablets. Adv. Pharm. Bull..

[35] Zhou H., Wan J., Wu L., Yi T., Liu W., Xu H., Yang X. (2013). A new strategy for enhancing the oral bioavailability of drugs with poor water-solubility and low liposolubility based on phospholipid complex and supersaturated SEDDS. PLoS ONE.

[36] Dyminski D., Paterno L., Takeda H., Bolini H., Mattoso L., Cândido L. (2006). Correlation between human panel and electronic tongue responses on the analysis of commercial sweeteners. Sens. Lett..

[37] Zhang X., Zhang Y., Meng Q., Li N., Ren L. (2015). Evaluation of beef by electronic tongue system TS-5000Z: Flavor assessment, recognition and chemical compositions according to its correlation with flavor. PLoS ONE.

[38] Holliday D.L., Speirs V. (2011). Choosing the right cell line for breast cancer research. Breast Cancer Res..

[39] Siddique A., Ibrahim H.Y., Akl M.R., Ayoub N.M., Goda A.A., Mohyeldin M.M., Nagumalli S.K., Hananeh W.M., Liu Y.Y., Meyer S.A. (2019). (-)-Oleocanthal combined with lapatinib treatment synergized against HER-2 positive breast cancer *in vitro* and *in vivo*. Nutrients.

[40] Ahmad A. (2013). Pathways to breast cancer recurrence. ISRN Oncol..

[41] Shim H.J., Kim S.H., Kang B.J., Choi B.G., Kim H.S., Cha E.S., Song B.J. (2014). Breast cancer recurrence according to molecular subtype. Asian Pac. J. Cancer Prev..

[42] Ho-Yen C.M., Jones J.L., Kermorgant S. (2015). The clinical and functional significance of c-Met in breast cancer: A review. Breast Cancer Res..

[43] Ho-Yen C.M., Green A.R., Rakha E.A., Brentnall A.R., Ellis I.O., Kermorgant S., Jones J.L. (2014). c-Met in invasive breast cancer. Is there a relationship with the basal-like subtype?. Cancer.

[44] Samiee S., Berardi P., Bouganim N., Vandermeer L., Arnaout A., Dent S., Mirsky D., Chasen M., Caudrelier J.M., Clemons M. (2012). Excision of the primary tumor in patients with metastatic breast cancer: A clinical dilemma. Curr. Oncol..

[45] Pienta K.J., Robertson B.A., Coffey D.S., Taichman R.S. (2013). The cancer diaspora: Metastasis beyond the seed and soil hypothesis. Clin. Cancer Res..

[46] Comen E., Norton L., Massagu E.J. (2011). Clinical implications of cancer self-seeding. Nat. Rev. Clin. Oncol..

[47] Dutta S., Sengupta P. (2016). Men and mice: Relating their ages. Life Sci..

